# Chaos Caused by Different Cutoff Dates: Relative Age Effects and Redshirting in Collegiate Volleyball in the United States

**DOI:** 10.3390/sports13020053

**Published:** 2025-02-11

**Authors:** Grace Redman, Scott Pierce, Adam Leigh Kelly

**Affiliations:** 1 School of Kinesiology and Recreation, Illinois State University, Normal, IL 61761, USA; swpierc@ilstu.edu; 2 Research for Athlete and Youth Sport Development (RAYSD) Laboratory, Research Centre for Life and Sport Sciences (CLaSS), College of Life Sciences, Birmingham City University, Birmingham B15 3TN, UK; adam.kelly@bcu.ac.uk

**Keywords:** collegiate sport, RAE, NCAA, athletic timing, university sports club, school, training age

## Abstract

Relative Age Effects (RAEs) are a phenomenon in athletics related to an over-representation of individuals born closer to an arbitrary cutoff date. Such effects have been shown in many different countries, levels of play, and contexts, although they are yet to be studied in volleyball within the United States, which is the second most popular high school girls’ sport and the fastest growing high school and college sport for males. Therefore, the purpose of this study was to examine RAEs in college volleyball. Publicly available data were collected from the websites of women’s Division I program (*n* = 1253) and men’s Division I/II (*n =* 164). Chi-squared goodness of fit tests were used to compare birth rate distributions. Data accounted for gender, school and club cutoff dates, athletic timing, and redshirt status. Results showed RAEs were strongest in women on-time school group. Interestingly, reverse effects were observed (i.e., an overrepresentation of relatively younger athletes) for delayed school volleyball players, but this expected trend was not observed in the redshirt group. On-time women’s club group showed academic timing was a significant contributor towards RAEs, whilst these effects were strongest for the on-time school group in men.

## 1. Introduction

Grouping athletes by chronological age (i.e., U10, U11, U12, etc.) occurs at all levels of competition in youth sports. The aim of age grouping is to create an equal playing field where athletes are divided into fixed age groups [1]. Though the overall approach seems rational, it has unintentionally created a phenomenon commonly referred to as Relative Age Effects (RAEs). Relative Age Effects create a system whereby those who are born closer to an arbitrary cutoff date are overrepresented within age group rosters, whereas those born towards the end of the cutoff date are underrepresented [2,3,4]. This occurs due to a combination of factors [5]. For instance, those who are relatively older have had more time to grow and are often physically superior compared to the rest of their same age group peers [1,6]. Moreover, youth who are relatively older will have had the opportunity to train longer (i.e., an older training age) and subsequently developed advanced technical and tactical skills [7]. As a result, these performance-related characteristics are likely to impact upon social agents (i.e., parents, coaches, peers) perceptions of who they view as ‘talented’ (i.e., those who are relatively older), which can influence psychological and social attributes [8]. Whilst a 364-day difference may not seem like a lot on the surface, athletes who are relatively younger often negatively experience these effects [9].

The prevalence (i.e., the extent to which RAEs are prevailing) and coverage (i.e., the extent to which RAEs are present across different cohorts and contexts) of RAEs is generally consistent across different sports and contexts. The RAE has been evidenced in but not limited to the following sports: basketball [2], cricket [3], football [4], hockey [10], rugby [11], soccer [12], swimming [13], and tennis [14]. Although to varying degrees of magnitude, RAEs have been shown at all levels of competition ranging from youth sports [15], collegiate level sports [16], professional sports [17], and Olympic sports [18]. Finding ways in which to reduce RAEs is important–but there may be some positive factors related with being born towards the end of the cutoff year as well. For instance, the ‘underdog hypothesis’ suggests the advantages of being relatively younger during athlete development [19]. In the National Hockey League (NHL), for example, those born toward the end of a selection year, though underrepresented and drafted later, played in more games and scored more points when compared to those closer to the beginning of the selection year [20]. This higher productivity at the professional level could be due to developing greater technical and tactical skills, psychological characteristics, and/or physical capabilities over the long term due to being relatively younger (i.e., facing reoccurring challenges during youth) [21,22].

While much of the research related to RAEs has been conducted outside of the United States, some studies have explored its prevalence in the National Collegiate Athletic Association (NCAA) sports. For example, studies examining NCAA Division I Basketball [16], NCAA Division I Soccer [23], top NCAA golfers [24], and top-ranked Division I Softball [25] have found RAEs when accounting for academic timing (AT). Academic timing is based on specific school cutoff dates, and students’ entrance is categorized as ‘advanced’, ‘on-time’ (OT), or ‘delayed’ (D). Advanced entrance to school is when someone is not 5 years old at the cutoff date but still begins, which means they are younger than the majority of their cohort. On-time entrance is when someone is 5 years old by the appropriate cutoff date to kindergarten. Although delayed students are categorized as those who are old enough to enter kindergarten according to the cutoff date, they choose to wait to enter kindergarten until the following academic year. Notably, however, no relative age studies have been conducted in volleyball in the United States to test the impact of AT, despite being the second most popular girls’ high school sport based on total number of participants and being the top girls’ sport in 21 states [26], as well as one of the fastest-growing high school and college sport for boys [27].

Globally, research on RAEs in volleyball is generally mixed. For example, some studies that focused on adolescence volleyball players (aged 13–14 years) showed RAEs were prevalent in both male and female cohorts [28,29]. However, Rubajczyk and colleagues [29] revealed RAEs did not have a distinct effect on performance variables in 14-year-old boys and girls who participated in the Olympic Hope Tournament. Similarly, Okazaki and colleagues [30] observed RAEs in 11–14-year-old females participating in the International Volleyball Cup competition. Specifically, the authors observed sport competition anxiety as a better predictor of RAEs when compared to body composition and height. Contrastingly, however, other studies on 13- and 14-year-old girls did not observe RAEs. This includes Ntozis and colleagues [31], who looked at 13-to-14-year-olds who participated in the national age group selected training camps in Athens (Greece), as well as Papadopoulou and colleagues [32], who looked at 13- and 14-year-olds who were part of volleyball clubs in Athens (Greece).

Other studies have researched university-aged volleyball players (aged 18–22 years). For example, the first study into RAEs from Grondin and colleagues [33] in 1983 showed no effects in Canadian volleyball players (aged 17–19 years), although they showed it was prevalent in ice hockey. Similarly, Lidor and colleagues [34] studied Israeli university female volleyball players and did not observe any RAEs. More recently, however, Campos and colleagues [35] showed RAEs in under-18 volleyball players, with higher performing teams having more pronounced RAEs. Moreover, Safranyos and colleagues [36] studied RAEs over an eight-year period within university volleyball in men and women. They revealed significant differences in birth rate distribution for five of the eight years for men and six of the eight years for women. When accounting for athletic timing, the OT men and women’s data had significant differences in birth rate distribution seven out of the eight years. Specifically, the OT women had significantly overrepresented birth quarter one (i.e., those born in the first three month of the selection year; Q1), Q2, and partially Q3, and underrepresented Q4 (i.e., those born in the last three months of the selection year), and the OT men had significantly overrepresented Q1 and Q2 and underrepresented Q3 and Q4.

At the professional level of volleyball, there are mixed findings as well. Parma and colleagues [37] showed RAEs when looking at Brazilian Volleyball Confederation in men but not in women, whereas de Oliveira Castro and colleagues [38] also found RAEs were present in professional men’s professional volleyball, whereas in women’s volleyball it was only seen in the second highest division (Superliga B) and not in the highest division (Superliga A). Meanwhile, Nakata and colleagues [39] found the opposite in Japanese athletes, whereby there were RAEs observed in women but not in men. As another example, de Oliveira Castro and colleagues [40] investigated more contextual factors of RAEs in volleyball, including playing position and performance indicators. They showed RAEs in men who played the positions of outside hitter, opposite hitter, and middle hitters but did not find anything in liberos or setters. In comparison, females at this level showed no RAEs when grouped together, based on position, or through low/high performance. In the 2016 Olympics, however, Solon and Silva [41] observed no RAE in the males who scored (earned a point attacking, serving, or blocking). Taken together, the relative age findings remain varied in volleyball, both within and across genders, and should thus not be generalized to other contexts.

Examining the sport of volleyball in the NCAA within the United States is unique to previous relative age studies, as participants are impacted by two different cutoff dates. First, while most relative age studies include athlete participants who are only affected by one cutoff date (e.g., 1 January), an NCAA volleyball athlete from the United States is impacted by a ‘club cutoff date’ and an ‘school cutoff date’. The ‘club cutoff date’ is universal in the United States and refers to the last birthdate to begin participating in a youth club sport age group. The ‘school cutoff date’ is dependent on the state or town the students live in and refers to the last birthdate to begin participating in an academic/kindergarten class. To be specific, the kindergarten entrance requirement is that a child must be 5 years old by a particular date, which range from July 1st to January 1st (see Table 1). Plus, some states leave it up to the local government to decide that cutoff date which could expand to the list in Table 1. Studies on RAEs in other NCAA sports (e.g., soccer, golf) have used an estimated school cutoff date for all of the athletes without considering the state in which they started kindergarten [23,24] or did not consider the school cutoff date at all [16,25]. Though, as proven above when looking at RAEs, AT needs to be considered in colligate sports [16].

A second unique consideration for this athlete population is ‘redshirting’. Redshirting is very similar to holding an athlete back in kindergarten as it is delaying one’s entrance into collegiate athletics, potentially looking at redshirting as a means to even the playing field and give those who may be relatively younger an extra year to develop. Specifically, the NCAA has a ‘five-year rule’ for college athletes’ seasons of competition. Once an athlete starts their full-time academic program, they have five calendar years to compete in four seasons of intercollegiate competition. Redshirting means an athlete takes one year away from competition by choice or unwillingly, based on their academic standing or an injury [42]. A ‘redshirt freshman’, which is what this study is focused on, represents a first-year collegiate athlete and coach making the conscious decision not to compete. This first year, a redshirt freshman will only practice with the team and work on developing their skills in hopes to be better prepared for the next year where they may have a greater opportunity to compete. To our knowledge, however, there is no current research on collegiate redshirting and its relationship with RAEs in sport or volleyball.

## 2. Purpose

Exploring whether RAEs exist in collegiate volleyball is an important step to understand if they are apparent, particularly due to the different cutoff dates and redshirting rules in the United States. Thus, the purpose of this study was to examine RAEs in the Division I (DI) level of volleyball for women and Division I/II level of volleyball for men (these divisions are currently combined and compete against each other in the NCAA). More specifically, this study sought to determine (1) the distribution of DI women’s volleyball athletes based on school cutoff, accounting for AT and redshirting; (2) the distribution of DI women’s volleyball athletes based on club cutoff, accounting for AT and redshirting; (3) the distribution of DI/II men’s volleyball athletes based on school cutoff, accounting for AT and redshirting; and (4) the distribution of DI/II men’s volleyball athletes based on club cutoff, accounting for AT and redshirting.

Based upon the existing research presented, we hypothesized that (1) in women’s school volleyball, the birth quarter distribution of athletes would be Q1 > Q2 > Q3 > Q4 and these differences would be strongest in on-time athletes, while those who redshirted as freshmen or were delayed at kindergarten would have reverse effects; (2) AT would not be a significant contributor to RAEs for the women’s club group; (3) in men’s volleyball, the birth quarter distribution of athletes would be Q1 > Q2 > Q3 > Q4, and these differences would be the strongest in on-time athletes, while those who redshirted as freshmen or were delayed at kindergarten would have the reverse effects; and (4) AT would not be a significant contributor to RAEs for the men’s club group.

## 3. Materials and Methods

### 3.1. Participants

The sample consisted of 1417 Division I volleyball athletes, including 1253 women and 164 males. Secondary data from the 2020–2021 school year were collected from the official athletic department websites, including 116 out of the 334 Division I women’s volleyball programs (represented 37% all DI Schools) and 12 out of the 48 combined Division I and II Men’s volleyball programs (represented 25% of all DI/II schools). This represented all the publicly available data at these levels and included, at minimum, each athlete’s birth month and birthday (see Supplementary Data File).

### 3.2. Methods and Procedures

The minimum data feature collected for each individual was the athlete’s birth month. Birth day and year were included if they were available. The home state of each athlete was also recorded to allow for a comparison between each individual state and their specific school cutoff date, which varied based on states (see Table 1). If available, it was also noted if the athlete redshirted their freshman season (*n* = 34). All of the data collected were publicly available and used solely for the purpose of this study.

**Figure 1:**
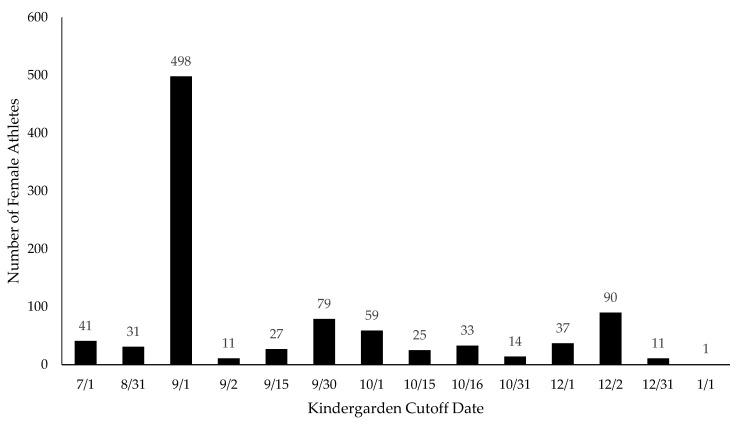
Division I Women’s Volleyball’s school cutoff distribution.

**Figure 2:**
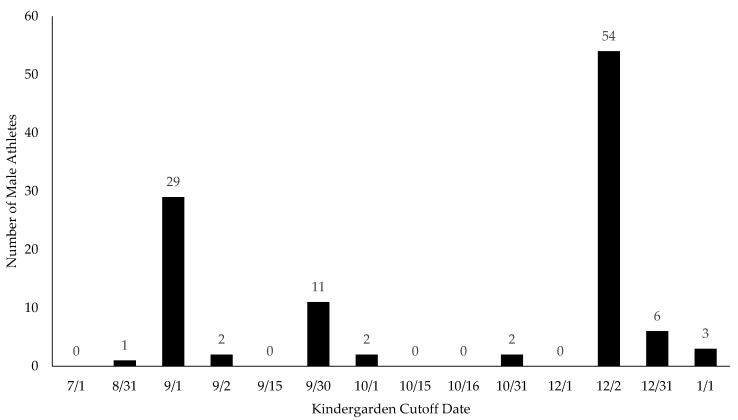
Division I/II Men’s Volleyball’s school cutoff distribution.

Conforming to the first strategy used in other RAE studies in the United States, the first approach was to divide up the subjects by birth month. There is a universal club cutoff date for club volleyball in the United States of 1 September [43]. The school volleyball cutoff date is dependent on each of the 50 state’s regulations for entering kindergarten, which range from turning 5 on 1 July–1 January [44]. For the club data, each player was assigned a birth quarter (BQ) corresponding to their birthdate to create an observed BQ distribution within each of the four cohorts (i.e., BQ1 = September, October, November; BQ2 = December, January, February; BQ3 = March, April, May; and BQ4 = June, July, August). The second approach used to group the athletes was based on their school cutoff date. The cutoff date was identified from the 2005 cutoff [44]. This was as close as could be found to the years the athlete would have entered kindergarten (97.3% of men and 99.1% of women were born between 1998 and 2002). Each athlete was put into the corresponding month related to their individual kindergarten cutoff date. Figure 1 and Figure 2 show the distribution of cutoff dates for women and men, respectively. The states which were considered LEA (left up to local education) or had multiple potential cutoff dates were not used in the school cutoff data analysis as there was no way to know the true cutoff date for these athletes. International students were excluded from these data, since their cutoff dates were unknown and were not the same as those in the United States. Graduate students were also excluded from the school cutoff data since their exact educational year could not be established.

The athletes were divided into two groups based on their AT. The first group was ‘on-time’, meaning they were within the appropriate birthdate ranges for their college class (freshman, sophomore, junior, senior) related to their specific states cutoff. The second group was ‘delayed’, where they were a year behind their class peers, which would make them chronologically older. The specific reason for this potential delay related to AT is unknown; it could be due to waiting to enter kindergarten, being held back a year, completing an extra year of high school, or delaying the start of college.

### 3.3. Statistical Analysis

A chi-squared (χ²) analysis was conducted using Microsoft Excel (Microsoft Corp., Redmond, WA, USA) to compare quartile distributions in the sample against the United States Center for Disease Control and Prevention (CDC) birthdate distribution of the years 1999 [45], 2000 [46], 2001 [47], and 2002 [48]. This test does not reveal the magnitude of the difference for significance, thus chi-squared and Cramer’s V were also used. Cramer’s V was interpreted as follows: a value of 0.06 or more indicates a small effect size, 0.17 or more indicates a medium effect size, and 0.29 or more indicates a large effect size [49]. Additionally, 95% confidence intervals (CIs) and odds ratio (OR) were used to compare quartiles.

## 4. Results

### 4.1. Women’s School

**Figure 3:**
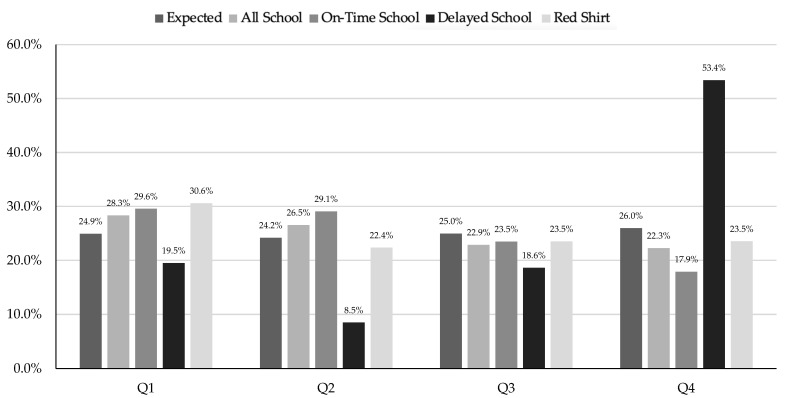
Division I women’s school cutoff by birth quartile.

Women’s combined school data (Table 2) showed significant differences between birth quartiles (X^2^ = 13.32, df = 3, *p* = 0.004, V = 0.083), with Q1 and Q2 overrepresented compared to Q4 (1.33 and 1.28, respectively). When dividing the groups by AT, there were significant differences between Qs for on-time athletes (X^2^ = 37.78, df = 3, *p* < 0.001, V = 0.150), with ORs between birth quartiles for Q1 vs. Q2, Q2 vs. Q4, and Q3 vs. Q4 showing 1.73, 1.75, and 1.37, respectively. The delayed athlete group was also significantly skewed (X^2^ = 50.59, df = 3, *p* < 0.001, V = 0.463), with ORs for Q1 vs. Q4, Q2 vs. Q4, and Q3 vs. Q4 being 0.38, 0.17, and 0.35, respectively. There were no significant differences between birth quartiles for the school cutoff redshirt group (X^2^ = 1.50, df = 3, *p* = 0.683, V = 0.094). The distribution of the women’s school cutoff by birth quartile is shown in Figure 3.

### 4.2. Women’s Club

**Figure 4:**
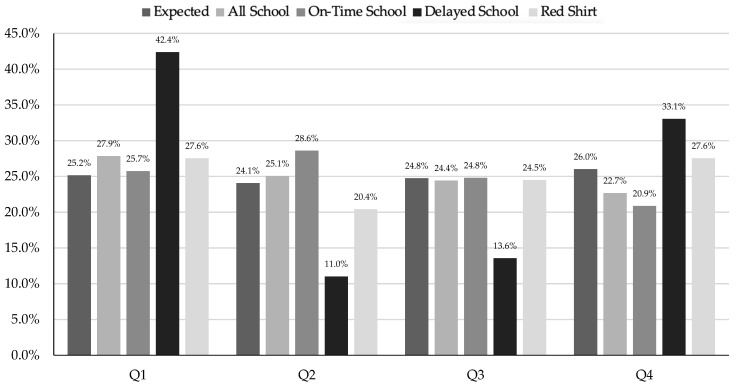
Division I women’s club cutoff by birth quartile.

Using the universal club cutoff of September 1st, there were significant differences between birth quartiles for the women’s club data (Table 2) (X^2^ = 9.6, df = 3, *p* = 0.022, V = 0.062). The largest difference between birth quartiles was Q1 vs. Q4, with an OR comparison of 1.27. When accounting for AT, there was a significant difference between birth quartiles for the OT club group (X^2^ = 15.88, df = 3, *p* <. 0.001, V = 0.097), with the largest difference for Q2 vs. Q4 (OR 1.48). There was also a significant difference between birth quartiles in the women’s club delayed group (X^2^ = 30.46, df = 3, *p* < 0.001, V = 0.359), with ORs for Q1 vs. Q4, Q2 vs. Q4, and Q3 vs. Q4 being 1.32, 0.36, and 0.43, respectively. There was no significance difference between birth quartiles in the women’s club redshirt group (X^2^ = 0.86, df = 3, *p* = 0.835, V = 0.066) The distribution of the women’s club cutoff by birth quartile is shown in Figure 4.

### 4.3. Men’s School

**Figure 5:**
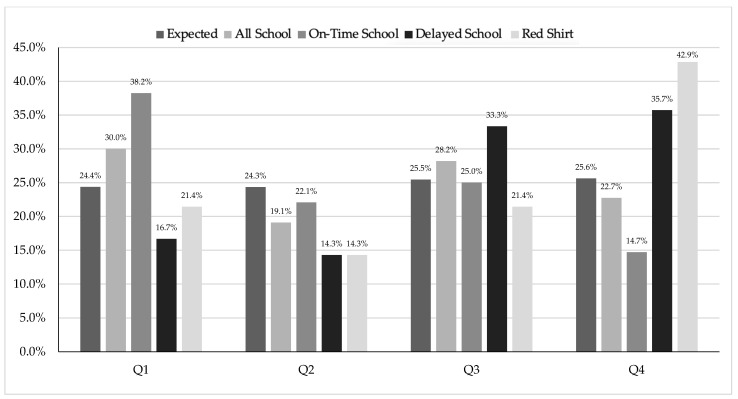
Division I/II men’s school cutoff by birth quartile.

When analyzing all the men’s school data (Table 3), there were no significant differences between birth quartiles (X^2^ = 3.77, df = 3, *p* = 0.288, V = 0.131). However, there were significant differences between birth quartiles for the OT school group (X^2^ = 8.99, df = 3, *p* = 0.029, V = 0.257), with the biggest difference between Q1 and Q4 (OR 2.80). In comparison, the men’s delayed group (X^2^ = 5.72, df = 3, *p* = 0.126, V = 0.261) and the men’s redshirt group (X^2^ = 2.29, df = 3, *p* = 0.513, V = 0.267) had no significant differences between birth quartiles The distribution of the men’s school cutoff by birth quartile is shown in Figure 5.

### 4.4. Men’s Club

**Figure 6:**
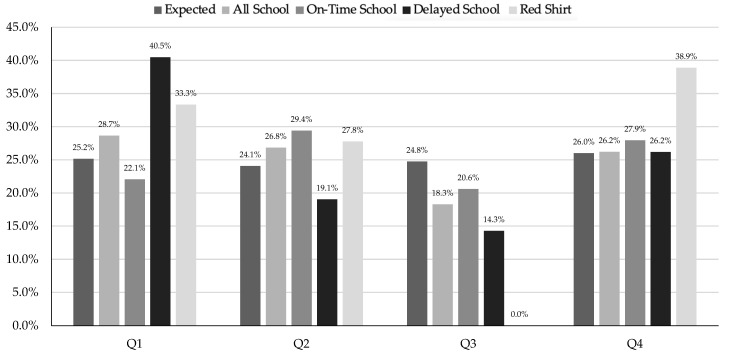
Division I/II men’s club cutoff by birth quartile.

The men’s club data had no significant differences between birth quartiles for the group combined (X^2^ = 4.08, df = 3, *p* = 0.253, V = 0.112) or when split into sub-groups, including club OT (X^2^ = 1.64, df = 3, *p* = 0.651, V = 0.110), club delayed (X^2^ = 6.21, df = 3, *p* = 0.102, V = 0.272), and club redshirt (X^2^ = 6.19, df = 3, *p* = 0.103, V = 0.415). The distribution of the men’s club cutoff by birth quartile is shown in Figure 6.

## 5. Discussion

As volleyball is a growing sport in both participation and popularity in the United States [26,27], it is important to examine and understand the developmental trajectories of youth volleyball players. To do this, the purpose of this study was to examine RAEs in the Division I level of volleyball for both men and women. We also wanted to better understand the impact of sixteen different school cutoff dates across the United States and how this could potentially impact RAEs based on a universal club cutoff date. When observing both club and school cutoff dates for women, there were significant RAE findings for women volleyball players. For both school and club cutoff dates, women volleyball players born earlier in the year were overrepresented in DI collegiate women’s volleyball. When comparing the women’s data and the two cutoff dates, the stronger significance consistently paired with the school cutoff groupings (all women and OT women). In comparison, there were only significant findings for the men’s OT school group.

Overall, our hypotheses were partially supported. First, RAEs in women were the strongest in the OT school group and the reverse effects for delayed school athletes were observed, but this trend was not apparent in the redshirt group. Second, contrary to our hypothesis, women’s OT club data showed AT was a significant contributor to RAEs. Third, RAEs in men was the strongest in the OT school group and, though not significant, the data showed the last two birth quartiles contained 69% of the athletes. Our hypothesis on men’s school redshirting had such a low sample size *(n* = 14) the hypothesis could not be proved or disproved. Fourth, as hypothesized for men, AT was not a significant contributor to RAEs related to the club cutoff.

### 5.1. Women’s School

The first sub-purpose of the study was to examine the distribution of DI women’s volleyball athletes based on school cutoff, accounting for AT and redshirting. The women’s school cutoff followed the expected trends of RAEs (i.e., Q1 > Q2 > Q3 > Q4) [50]. This was strongest in the on-time group. The second sub-purpose was to examine the distribution of DI women’s volleyball athletes based on club cutoff, accounting for AT and redshirting. The club data also followed the expected RAE trend, although it was not as strong. This is possibly due to the fact that the club cutoff was the same for 52% of the female athletes, and about 78% of the athletes’ school cutoff dates were within the first or second month of the club cutoff.

### 5.2. Women’s Club

When continuing to divide the female players into groups, the on-time school athletes also followed the expected relative age pattern but with stronger effects, which is what has been seen in other studies when accounting for AT [51]. Interestingly, delayed athletes have been shown to have the reverse effect where a larger percentage of athletes are born closer to the end of the cutoff years, as opposed to the start [52], which was consistent with our findings of delayed women’s school cutoff group athletes, which had over half of the group (53.39%) in the final quarter of the year. Both the club data for the OT and delayed athletes did not follow the expected trend. For the OT group, there was a higher-than-expected number of athletes in Q2, and the delayed group had the greatest percentage of the group in Q1 and Q4. The ‘Q2 Conundrum’ is something that has been seen in female athlete populations before, where Q2 representation has been found to be equal or greater to Q1, as well as greater than Q3 and Q4 [53].

Our study is unique in that it examined how young athletes may be affected by more than one cutoff date in their sport. For this population, chaos is caused by sixteen different school cutoff dates (as well as potentially many other unknown cutoff dates that are left up to the local education authority) and one club cutoff date for volleyball players. Conceptually, it would make sense that these cutoff dates would contribute to the variation in the distribution of players across birth quartiles for school and club volleyball, possibly helping to negate RAEs [54]. Additionally, as club volleyball is recognized as the most prominent domain for recruiting collegiate volleyball players, it may be assumed that the RAEs based on club cutoffs might be most relevant for the collegiate women’s population. It was notable, however, that RAEs were significant for women volleyball players using both school and club cutoff dates. This nuance and chaos caused by multiple cutoff dates require further examination in relative age research.

### 5.3. Men’s School

When examining the distribution of DI/II men’s volleyball athletes based on school cutoff, accounting for AT and redshirting, the only significant men’s group was the OT school. This aligned with the idea that the school may be the dominant cutoff date, though the sample size was small. Specifically, the descriptive data showed a skewed distribution, with Q1 (38.2%) having the most players and Q4 (14.7%) having the least. Interestingly, the overall BQ2 was slightly smaller than BQ3, which did not completely follow the expected pattern of Q1 > Q2 > Q3 > Q4, but for the small sample size, it showed the potential presence of RAEs. These men’s data are telling, as many have suggested using multiple cutoff dates to negate RAEs [54]. Almost half (49.1%) of the men had a cutoff date for school of December 2nd paired with a club cutoff date of July 1st, but the only significant data were paired with the school data.

### 5.4. Men’s Club

As there was no significant findings for DI/II men’s volleyball athletes based on club cutoff, accounting for AT or redshirting, as stated before, it is hard to draw any conclusions from these data outside of the potential of having a dominant cutoff date. Again, the small sample may make it difficult to see any RAE trends as well.

### 5.5. Redshirting

The lack of significance in redshirting for men and women across both school cutoffs and club cutoffs was not surprising based on the small sample size of athletes who redshirted their first year. Redshirting does not seem to be particularly common in volleyball. Specifically, only 11% of the men and 8% of the women from this sample redshirted. When continuing to research redshirting and its connection to RAEs, policy changes in collegiate sport and sport-specific contexts should be considered. For example, transferring programs can impact eligibility and may increase the number of ‘delayed’ athletes in the system. Furthermore, examining the relationship between RAEs and redshirting in the United States college football may be insightful. During the same season (2020–2021), the number one collegiate football team, Alabama, had a roster in which 50/131 (38.2%) redshirted at some point in their career according to their athletic website. This may be due to higher levels of competition, a wider pool of potential talent, more money, and/or more resources available.

### 5.6. Limitations and Future Direction

The main limitation of this study is the relatively small percentage of the total NCAA volleyball players who were included. Specifically, 34.7% of the total DI women’s and 25% of the total DI/DII men’s schools were included, and not every athlete from each of these included schools had available data. Indeed, the men’s data had a particularly small sample size, which makes it difficult to generalize these results. There were also no data for Division II or III women’s volleyball, as well as Division III men’s volleyball. This left out 81% of the women who played volleyball in college and 41% of the men who competed at the collegiate level [55]. Nevertheless, this study provides a useful opening to better understand the chaos caused by different cutoff dates, as well as the impact of redshirting on RAEs in collegiate volleyball in the United States.

This study only scratched the surface of RAEs in NCAA volleyball. Moving forward, collecting more data on these athletes, including physiological maturity, playing position, reason for attending university/college, and analyzing data regionally for both where these athletes grew up and where they went to university/college. When it comes to redshirting, collecting more data as to why they redshirted, who made the decision, and potentially their performance variables over all their years of competition may help to have more complete understanding of the phenomenon and how it may connect with RAEs.

## 6. Conclusions

No matter the confusion caused by the numerous cutoff dates for school in the United States, RAEs are prevalent in men and women’s volleyball when accounting for the school cutoff and athletic timing. This provides an unfair advantage to those born closer to their state’s cutoff date. Though these individuals are affected by two dates (club and school), our data suggested the following: (1) Women’s school group did follow the expected RAE pattern of Q1 > Q2 > Q3 > Q4, with the on-time athletes having stronger RAEs. There was no significance associated with redshirting. (2) The on-time club group showed AT was a significant contributor to RAEs. (3) RAE in the school men’s group was only significant for the OT men. There was no significance associated with redshirting (4) There was no significant findings related to the club cutoff. Overall, the school cutoff date seemed to be predominant, as shown by the fact that about half of the men were affected by two cutoff dates five months apart, but only RAEs related to the school cutoff date were present. Additionally, women were found to have stronger significant distributions when comparing the school and club data as a whole and for OT athletes. It is important to acknowledge there may be unintended consequences when adapting the existing age group structures; thus, it is important to ensure that such attempts are evaluated before being widely implemented. That being said, continuing to investigate ways to reduce RAEs, including redshirting, is necessary. For volleyball to continue to grow in popularity in the United States and around the world, the goal should be to even the playing field at early ages for all who want to be involved in the sport.

## Figures and Tables

**Table 1:** Individual state academic cutoff dates.

Cutoff Date	States
1-July	Indiana
1-August	Missouri
15-August	Alaska
31-August	Delaware, Kansas, Washington
1-September	Alabama, Arizona, Florida, Georgia, Idaho, Illinois, Minnesota, Mississippi, New Mexico, North Dakota, Oklahoma, Oregon, Rhode Island, South Carolina, South Dakota, Texas, West Virginia, Wisconsin
2-September	Utah
15-September	Arizona, Iowa, Wyoming
30-September	Louisiana, Nevada, Tennessee, Virginia
1-October	Colorado, Kentucky
15-October	Maine, Nebraska
16-October	North Carolina
31-October	Maryland
1-December	Michigan
2-December	California
31-December	District of Columbia, Hawaii
1-January	Connecticut
LEA^1^	Massachusetts, New Hampshire, New Jersey, New York, Pennsylvania, Vermont, Montana, Ohio

^1^ LEA: Decision on cutoff date is left up to the Local Education Agency.

**Table 2:** The Division I Women’s Volleyball’s birth quartile distribution.

	Q1	Q2	Q3	Q4	Total	X2	*p*	Cramer’s V	Odds Ratio Comparisons (95% Confidence Intervals)		
									Q1 vs. Q4	Q2 vs. Q4	Q3 vs. Q4
School	271	254	219	213	957	13.32	**0.004**	0.083	1.33	1.28	1.07
	(28.32%)	(26.54%)	(22.88%)	(22.26%)					(1.0–1.7)	(1.0–1.7)	(0.8–1.4)
Club	349	314	306	284	1253	9.6	**0.022**	0.062	1.27	1.20	1.12
	(27.85%)	(25.06%)	(24.42%)	(22.67%)					(1.0–1.6)	(1.0–1.5)	(0.9–1.4)
School on-time	248	244	197	150	839	37.78	**<0.001**	0.150	1.73	1.75	1.37
	(29.56%)	(29.08%)	(23.48%)	(17.88%)					(1.3–2.3)	(1.3–2.3)	(1.0–1.8)
Club on-time	216	240	208	175	839	15.88	**0.001**	0.097	1.28	1.48	1.25
	(25.74%)	(28.61%)	(24.79%)	(20.86%)					1.0–1.7	(1.1–1.9)	(0.9–1.6)
School delayed	23	10	22	63	118	50.59	**<0.001**	0.463	0.38	0.17	0.35
	(19.49%)	(8.47%)	(18.64%)	(53.39%)					(0.2–0.8)	(0.1–0.4)	(0.2–0.7)
Club delayed	50	13	16	39	118	30.46	**<0.001**	0.359	1.32	0.36	0.43
	(42.37%)	(11.02%)	(13.56%)	(33.05%)					(0.7–2.6)	(0.2–0.8)	(0.2–0.9)
Redshirt school	26	19	20	20	85	1.5	0.683	0.094	1.36	1.00	1.04
	(30.59%)	(22.35%)	(23.53%)	(23.53%)					(0.6–3.1)	(0.4–2.4)	(0.4–2.5)
Redshirt club	27	20	24	27	98	0.86	0.835	0.066	1.03	0.80	0.93
	(27.55%)	(20.41%)	(24.49%)	(27.55%)					(0.5–2.2)	(0.4–1.8)	(0.4–2.0)

Bold signifies significance of *p* < 0.05.

**Table 3:** Division I/II Men’s Volleyball’s birth quartile distribution.

Cohort	Q1	Q2	Q3	Q4	Total	X2	*p*	Cramer’s V	Odds Ratio Comparisons (95% Confidence Intervals)		
									Q1 vs. Q4	Q2 vs. Q4	Q3 vs. Q4
School	33	21	31	25	110	3.77	0.288	0.131	1.39	0.85	1.25
	(30.00%)	(19.09%)	(28.18%)	(22.73%)					(0.7–2.9)	(0.4–1.9)	(0.6–2.6)
Club	47	44	30	43	164	4.08	0.253	0.112	1.13	1.11	0.73
	(28.66%)	(26.83%)	(18.29%)	(26.22%)					(0.6–2.0)	(0.6–2.0)	(0.4–1.4)
School on-time	26	15	17	10	68	8.99	**0.029**	0.257	2.80	1.61	1.76
	(38.24%)	(22.06%)	(25.00%)	(14.71%)					(1.0–7.5)	(0.6–4.6)	(0.6–4.9)
Club on-time	15	20	14	19	68	1.64	0.651	0.110	0.82	1.14	0.77
	(22.06%)	(29.41%)	(20.59%)	(27.94%)					(0.3–2.1)	(0.5–2.9)	(0.3–2.0)
School delayed	7	6	14	15	42	5.72	0.126	0.261	0.48	0.41	0.92
	(16.67%)	(14.29%)	(33.33%)	(35.71%)					(0.1–1.7)	(0.1–1.5)	(0.3–2.8)
Club delayed	17	8	6	11	42	6.21	0.102	0.272	1.60	0.79	0.57
	(40.48%)	(19.05%)	(14.29%)	(26.19%)					(0.5–5.0)	(0.2–2.7)	(0.2–2.1)
Redshirt school	3	2	3	6	14	2.29	0.513	0.267	0.53	0.35	0.51
	(21.43%)	(14.29%)	(21.43%)	(42.86%)					(0.1–4.1)	(0.0–3.1)	(0.1–3.8)
Redshirt club	6	5	0	7	18	6.19	0.103	0.415	0.89	0.77	0.00
	(33.33%)	(27.78%)	(0.00%)	(38.89%)					(0.2–4.8)	(0.1–4.4)	(0.0–1.7)

Bold signifies significance of *p* < 0.05.

## Data Availability

The data presented in this study are available on request from the corresponding author and will be made available without reservation.

## Supplementary Materials

The following are available online at https://www.mdpi.com/article/10.3390/sports13020053/s1, Table S1: Supplementary Data File.
